# Collecting duct cells show differential retinoic acid responses to acute versus chronic kidney injury stimuli

**DOI:** 10.1038/s41598-020-73099-9

**Published:** 2020-10-07

**Authors:** Alexandros Papadimitriou, Paola Romagnani, Maria Lucia Angelotti, Mazhar Noor, Jonathan Corcoran, Katie Raby, Patricia D. Wilson, Joan Li, Donald Fraser, Remi Piedagnel, Bruce M. Hendry, Qihe Xu

**Affiliations:** 1 Renal Sciences and Integrative Chinese Medicine Laboratory, Department of Inflammation Biology, School of Immunology and Microbial Sciences, Faculty of Life Sciences and Medicine, King’s College London, London, UK; 2 Department of Clinical and Experimental Biomedical Sciences, University of Florence, Florence, Italy; 3 The Wolfson Centre for Age-Related Diseases, King’s College London, London, UK; 4 University College London, UCL Centre for Nephrology, Royal Free Hospital, London, UK; 5 Faculty of Medicine, University of Queensland, Brisbane, QLD Australia; 6 Wales Kidney Research Unit, Heath Park Campus, Cardiff, UK; 7 National Institute for Health and Medical Research (INSERM), Unité Mixte de Recherche (UMR)-S1155, Tenon Hospital, Sorbonne Universités, Paris, France

**Keywords:** Acute kidney injury, Chronic kidney disease, Polycystic kidney disease, Toxin-induced nephropathy, Cell signalling, Drug discovery

## Abstract

Retinoic acid (RA) activates RA receptors (RAR), resulting in RA response element (RARE)-dependent gene expression in renal collecting duct (CD). Emerging evidence supports a protective role for this activity in acute kidney injury (AKI) and chronic kidney disease (CKD). Herein, we examined this activity in *RARE-LacZ* transgenic mice and by RARE-Luciferase reporter assays in CD cells, and investigated how this activity responds to neurotransmitters and mediators of kidney injury. In *RARE-LacZ* mice, Adriamycin-induced heavy albuminuria was associated with reduced RA/RAR activity in CD cells. In cultured CD cells, RA/RAR activity was repressed by acetylcholine, albumin, aldosterone, angiotensin II, high glucose, cisplatin and lipopolysaccharide, but was induced by aristolochic acid I, calcitonin gene-related peptide, endothelin-1, gentamicin, norepinephrine and vasopressin. Compared with age-matched normal human CD cells, CD-derived renal cystic epithelial cells from patients with autosomal recessive polycystic kidney disease (ARPKD) had significantly lower RA/RAR activity. Synthetic RAR agonist RA-568 was more potent than RA in rescuing RA/RAR activity repressed by albumin, high glucose, angiotensin II, aldosterone, cisplatin and lipopolysaccharide. Hence, RA/RAR  in CD cells is a convergence point of regulation by neurotransmitters and mediators of kidney injury, and may be a novel therapeutic target.

## Introduction

Acute kidney injury (AKI) is an abrupt renal insult followed by sudden decline of kidney functions^1,2^; chronic kidney disease (CKD) is a long-term condition where the kidneys do not work effectively, resulting in chronic loss of renal functions^3,4^. AKI occurs in 10–15% hospitalised patients and approximately 50% patients in intensive care, and is associated with high mortality^2^; affecting 8–16% of the world’s population^3,4^, CKD was the 12th most common cause of mortality and caused 4.6% of deaths globally in 2017^5^. If this rising prevalence of CKD continues, it is predicted that CKD will become a top-5 cause of mortality worldwide by 2040^6^.

AKI and CKD are not distinct disease entities but rather are closely interconnected syndromes^7^. Despite some shared aetiologies, risk factors and mechanisms, the pathophysiology of AKI and CKD can be quite different. Although AKI may be caused by systemic, vascular, glomerular and tubular diseases, the core pathophysiology of AKI is often predominated by tubulointerstitial injury^2^. On the other hand, although a wide range of aetiologies may all cause CKD, the latter is most often a glomerular disease^4^. However, regardless of the primary aetiologies of CKD, tubulointerstitial injury plays a crucial role in CKD progression to end-stage kidney disease^8^. Thus, it is critical to investigate the mechanism of tubulointerstitial injury, which is as yet poorly understood. What we do know, however, is that tubulointerstitial injury in AKI and CKD is associated with a wide range of overlapping aetiologies, risk factors and mediators. For example, infection, nephrotoxins and renal ischaemia–reperfusion contribute to tubulointerstitial injury in both AKI and CKD; while persistent albuminuria, diabetes and hypertension primarily cause CKD progression, although they may also contribute to increased risk of AKI^1–4,7^.

Endeavouring to better understand the mechanisms of tubulointerstitial injury, we have recently put forward a novel hypothesis that the canonical vitamin A signalling mediated by retinoic acids (RA) and retinoic acid receptors (RAR) in the renal collecting duct (CD) is a critical protector against renal injury and that mediators of kidney injury might contribute to tubulointerstitial injury by differentially regulating the RA/RAR activity in CD cells^9^. We have chosen to study the CD because emerging evidence suggests that the CD might play important roles in defence against infection^10^ and tubulointerstitial injury^11^, and might be involved in gentamicin- and ischaemia–reperfusion-induced AKI^12,13^ and obstructive CKD^14^. Furthermore, the CD meets many criteria for a key defence role in the kidney: it is in the right location to play a principal defence role—the CD is the only tubular structure that spans much of the kidney, ideally placed to protect the tubulointerstitial compartment; and it is equipped with defending cells and molecules: mesenchymal stem cells, antifibrotic miRNAs and the RA/RAR signalling^9^.

The activity of RA, especially all-*trans* RA (atRA), is mainly mediated through binding and activating heterodimers of RAR and retinoid X receptors (RXRs), thereby modulating gene transcription^15,16^. RARs and RXRs each have three isotypes (α, β and γ) with overlapping functions. Target genes of atRA are often characterised by the presence of one or more retinoic acid response elements (RAREs) in their gene regulatory regions, which serve as anchorage points for the RAR-RXR heterodimers^17,18^. The RAR-RXR heterodimers can be readily activated by RAR agonists, principally atRA, but cannot be activated by RXR agonists alone^19^. Thus, for convenience, the atRA-dependent RAR-RXR transcriptional activity is also widely referred to as the RA/RAR activity.

RARs and atRA are indispensable for embryonic kidney development, and even mild gestational vitamin A deficiency leads to a deficit in nephron number^20^. Using young and adult *RARE-LacZ* mice as a model to report endogenous RA/RAR activity, we have previously shown that the endogenous renal RA/RAR activity is physiologically confined to the CD^21^. Moreover, in embryonic kidneys after E11, the RA/RAR activity is confined to precursors of the CD known as ureteric bud (UB)^22^, so the UB/CD cell lineage appears to be the main site of RA/RAR activation in healthy kidneys.

To catalogue RA/RAR-dependent genes in CD cells in vitro, we first looked at mIMCD-3 cells, a well-characterised CD cell line derived from the mouse inner medulla^23,24^, which has been widely used in studying ion channel signalling^25,26^, signalling during osmotic and hypertonic stress^27^, urea signalling^28^ and branching morphogenesis^29^ of CD cells. We found that, similar to the CD, mIMCD-3 cells had significant endogenous RA/RAR activity and this activity was required for expression of a panel of RA/RAR-dependent genes, many of which are implicated in protection against bacterial infection, inflammation and fibrosis (*Ppbp*, *Lcn2*, *Bmp7*)^23^. Further supporting a renoprotective role for RA/RAR, vitamin A deficiency has been associated with increased susceptibility to pyelonephritis, urolithiasis, kidney inflammation and fibrosis^19,30–33^. On the other hand, vitamin A and RA treatment have well-documented therapeutic effects in infectious and non-infectious kidney diseases^19,30,34–38^ and the endogenous RA/RAR signalling in the kidney has been reported to be protective in ischaemia–reperfusion models of AKI in mice^39^, although the exact change and role for the RA/RAR in the CD remain un-answered.

To further examine our hypothesis about the role for the RA/RAR in the CD in tubulointerstitial injury in AKI and CKD, this study was designed to address whether and how neurotransmitters and mediators of kidney injury affect RA/RAR activity in CD cells. RA/RAR activity in CD cells was examined in *RARE-LacZ* mice^40^ and was quantified by RARE-Luciferase reporter assays in CD-derived cell cultures exposed to neurotransmitters and mediators of kidney injury, with or without atRA or RA-568, a selective agonist of RARα^41^, the most abundant RAR isotype in the kidney (Supplementary Figs. 1 and 2). Our results show that the RA/RAR activity in CD cells is a shared point of regulation by neurotransmitters and mediators of kidney injury and may be a potential target for clinical intervention by interacting drugs such as RA-568.

## Results

### RA/RAR activity in CD cells was repressed by albumin

**Figure 1:**
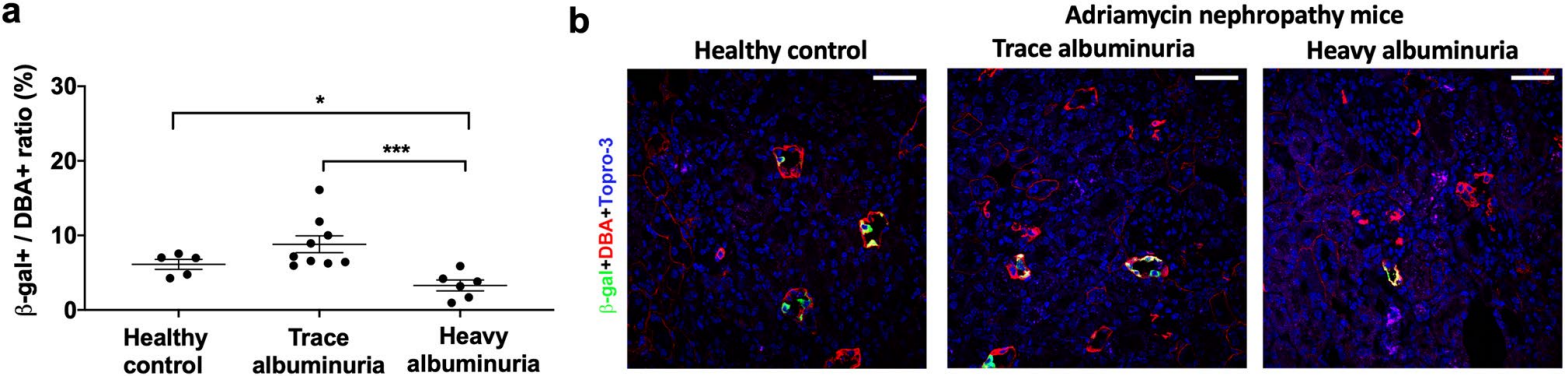
In Adriamycin nephropathy of *RARE-LacZ* mice, heavy albuminuria was associated with repressed RA/RAR activity. (**a**) RA/RAR activity in CD cells as a function of *RARE-LacZ* expression was reported as the ratio between number of β-gal-expressing CD cells and total number of DBA positive CD cells. Heavy albuminuria was associated with a 2.6-fold reduction of RA/RAR activity in CD cells; (**b**) Representative images of kidney tissues stained with an anti–β-galactosidase antibody (green), DBA-lectin (red, CD cells) and Topro-3 (blue, nuclei). In healthy control *RARE-LacZ* mice, RA/RAR activity (green signal) was physiologically confined to CD cells. In Adriamycin nephropathy mice, heavy albuminuria was associated with reduced RA/RAR activity in CD cells. *, ***: p < 0.05, p < 0.001, respectively.

**Figure 2:**
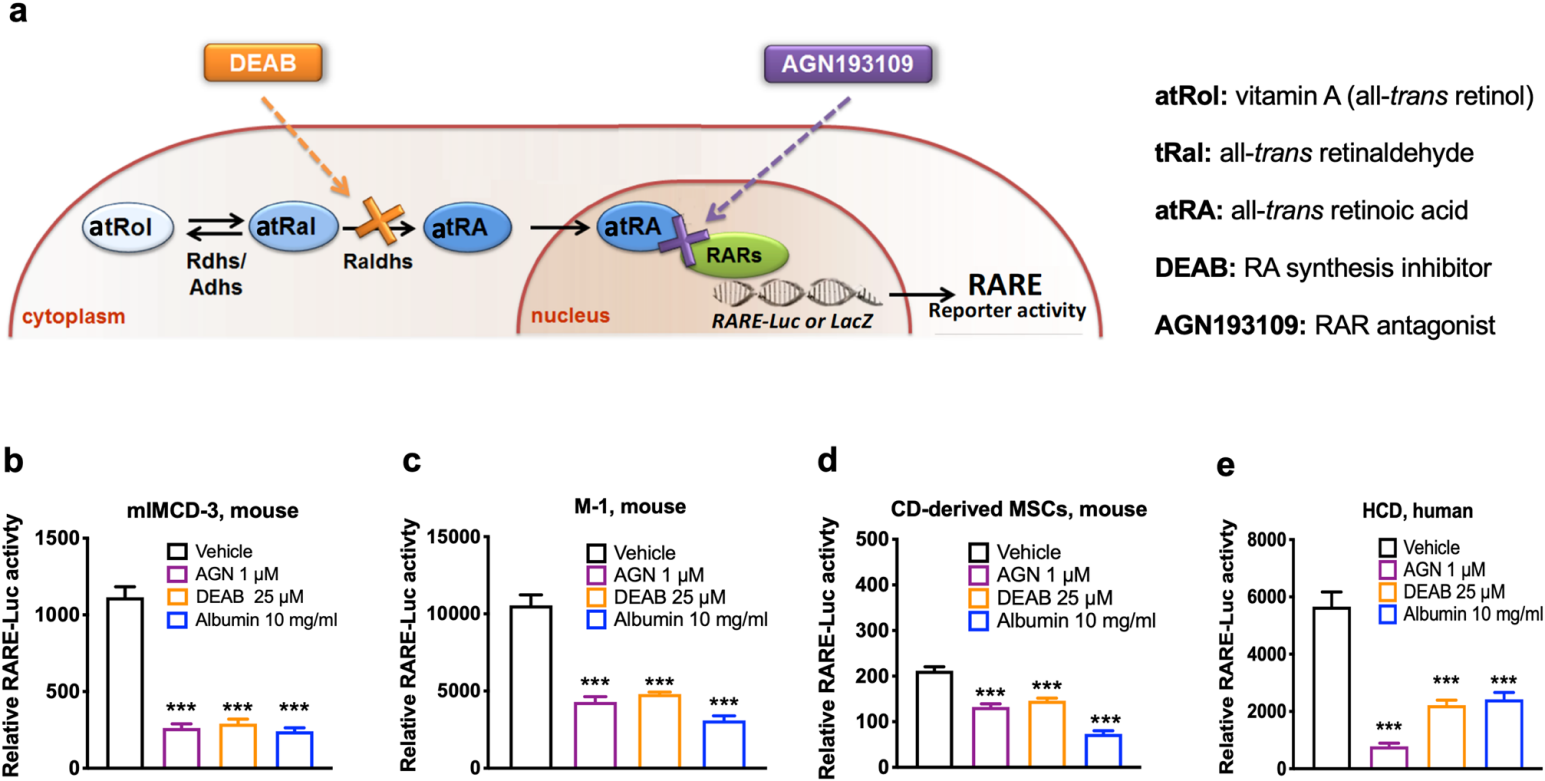
Mouse CD cells, CD-derived MSCs and human CD cells had endogenous RA/RAR activity, which was repressed by AGN193109, DEAB and albumin. (**a**) RA/RAR activity was defined as *RARE-LacZ* or *RARE-Luciferase* reporter activity. In cells, all-*trans* retinol (atRol), i.e., vitamin A, is oxidised reversibly to all-*trans* retinaldehyde (atRal), catalysed by the enzymes retinol dehydrogenases (Rdhs) or alcohol dehydrogenases (Adhs). atRal is then oxidised irreversibly to all-*trans* RA (atRA), catalysed by retinaldehyde dehydrogenases (Raldhs). atRA translocates into cell nucleus to bind and activate RARs, modulating gene transcription. AGN193109 (purple) was used to antagonise RAR. DEAB (orange) was used to inhibit Raldh-mediated atRA synthesis. In mIMCD-3 cells (**b**), M-1 cells (**c**) Hoxb7 B2 mouse CD-derived MSCs (**d**) and HCD cells (**e**), 1 μM AGN193109, 25 μM DEAB or 10 mg/ml albumin treatment for 24 h resulted in significant repression of RA/RAR activity. ***: *p* < 0.001 vs Vehicle control group.

**Figure 3:**
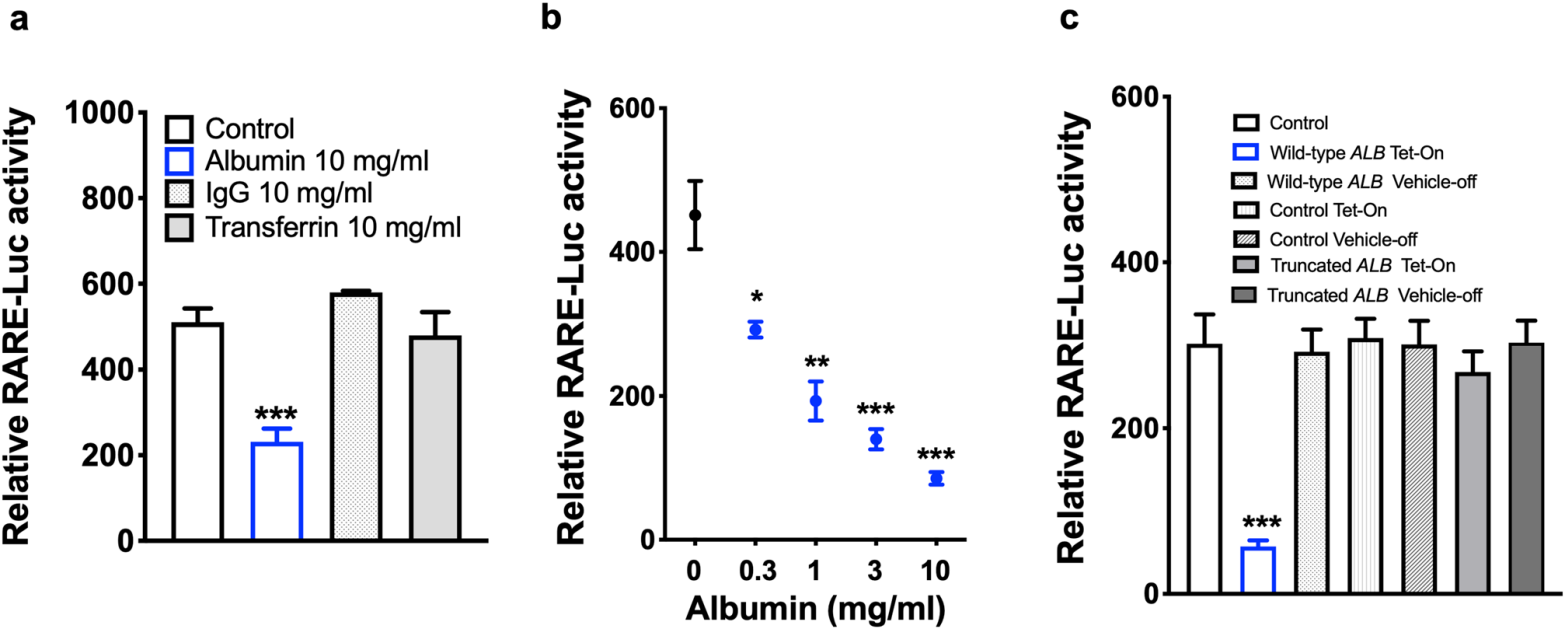
Albumin specifically and dose-dependently repressed RA/RAR activity. In mIMCD-3 cells, 24 h exposure to 10 mg/ml albumin, but not IgG or transferrin, repressed RA/RAR activity (**a**); Albumin repression of RA/RAR activity was dose-dependent (**b**); overexpression of *ALB*, but not *ALB* truncated of its RA-binding domain, repressed RA/RAR activity (**c**). *, **, ***: *p* < 0.05–0.001 vs Control, Vehicle-treated group. Tet-On, transgene induced by tetracycline; Vehicle-off, transgene silent in the presence of vehicle only.

Persistent albuminuria is a leading risk factor for CKD progression and reducing albuminuria has been associated with renoprotection in CKD^3,4,42^. We have previously reported that renal RA/RAR activity is physiologically confined to CD cells in *RARE-LacZ* mice^21^. We further found that, in Adriamycin-induced nephropathy, RA/RAR activity is pathologically switched on in glomerular progenitor cells to drive podocyte regeneration; however, when albuminuria becomes heavy, albumin reabsorbed by glomerular progenitor cells sequesters intracellular RA and dose-dependently represses RA/RAR activity, thus blocking podocyte regeneration^40^. Recognising that CD cells are known to re-absorb albumin^43,44^ and the latter is known to specifically bind atRA^45^, we examined whether albuminuria may lead to repressed RA/RAR activity in the CD. We found that, in *RARE-LacZ* mice, Adriamycin treatment induced heterogenous phenotypes in terms of severity of albuminuria. In those manifesting trace albuminuria, there was an insignificant trend towards increase of RA/RAR activity in the CD; in contrast, those manifesting heavy albuminuria were associated with significantly repressed RA/RAR activity in CD cells (Fig. 1a,b). We propose that RA/RAR in the CD may be repressed by albuminuria, but oppositely regulated by other mediators of injury and repair, leading to biphasic changes. To determine the exact relation between albumin and endogenous RA/RAR activity in CD cells and CD-derived cells, we examined the effects of albumin on this activity, with the following RA/RAR-repressing controls: (i) 4-(diethylamino)-benzaldehyde (DEAB), an inhibitor of retinaldehyde dehydrogenases (Raldhs), which inhibits endogenous atRA biosynthesis^21,46^; (ii) 4-[2-[5,6-Dihydro-5,5-dimethyl-8-(4-methylphenyl)-2-naphthalenyl]ethynyl]-benzoic acid (AGN-193109), a pan-RAR antagonist^23,47^. As we reported previously, endogenous RA/RAR activity was defined as RARE dual luciferase reporter activity repressed by both 25 μM DEAB and 1 μM AGN193109^23^ (Fig. 2a). In the mIMCD-3 mouse inner medullary CD cell line^23^ (Fig. 2b) and the M-1 mouse cortical CD cell line^48^ (Fig. 2c), CD-derived Hoxb7 B2 mesenchymal cells (MSCs)^49^ (Fig. 2d), as well as a human cortical CD cell line^50^ (Fig. 2e), clear endogenous RA/RAR activity repressed by both DEAB and AGN193109 was detected. In all these CD-derived cell cultures, 10 mg/ml albumin significantly repressed RA/RAR activity. In mIMCD-3 cells, we found that 10 mg/ml albumin, but not IgG and transferrin, repressed RA/RAR activity, indicating albumin specifically repressed RA/RAR activity (Fig. 3a). The effect of albumin was also dose-dependent at clinically relevant concentrations ranging from 0.3 mg/ml (equivalent to microalbuminuria) to 10 mg/ml (equivalent to heavy albuminuria) (Fig. 3b). To further confirm the specificity of albumin in repressing RA/RAR in CD cells and to shed light into the underlying mechanism, we found that overexpression of wild-type *ALB*, but not the gene truncated of the sequence encoding its RA-binding domain^40^, significantly repressed the endogenous RA/RAR activity (Fig. 3c). These data indicate that albumin excreted into urine could be reabsorbed by CD cells, leading to sequestration of RA and repression of RA/RAR activity in the cells, similar to what we reported previously in glomerular progenitor cells^40^.

Although no RA/RAR activity was detected in any non-CD cells in the healthy kidney in *RARE-LacZ* mice^21,22^, we do not rule out the possibility that non-CD renal cells have functional RA/RAR machinery that is physiologically below the detection limit of X-gal assay for reporting RA/RAR-dependent expression of *LacZ* gene^21^. Using in vitro cellular models and quantitative RARE dual luciferase reporter assays, we found that cultured mouse and rat proximal tubular cell lines (mPTEC and NRK-52E) and rat renal fibroblasts (NRK-49F) had endogenous RA/RAR activity, equivalent to 1/3–1/4 of the activity of mIMCD-3 cells. As in mIMCD-3 cells, the RA/RAR activity in these non-CD cell lines was repressed by DEAB, AGN193109, as well as albumin (Supplementary Figs. 3 & 4). Thus, although this project focuses on CD cells, some of our findings may also have implications for other relevant renal cell types. For example, physiologically, proximal tubules and the CD reabsorb 71% and 3% of albumin filtrated from glomeruli, respectively^51^. This means that albumin may exert stronger physiological repression on RA/RAR activity in proximal tubules, likely contributing to lack of visible physiological RA/RAR activity in these tubules in *RARE-LacZ* mice^21^.

### RA/RAR activity in CD cells was repressed by high glucose

**Figure 4:**
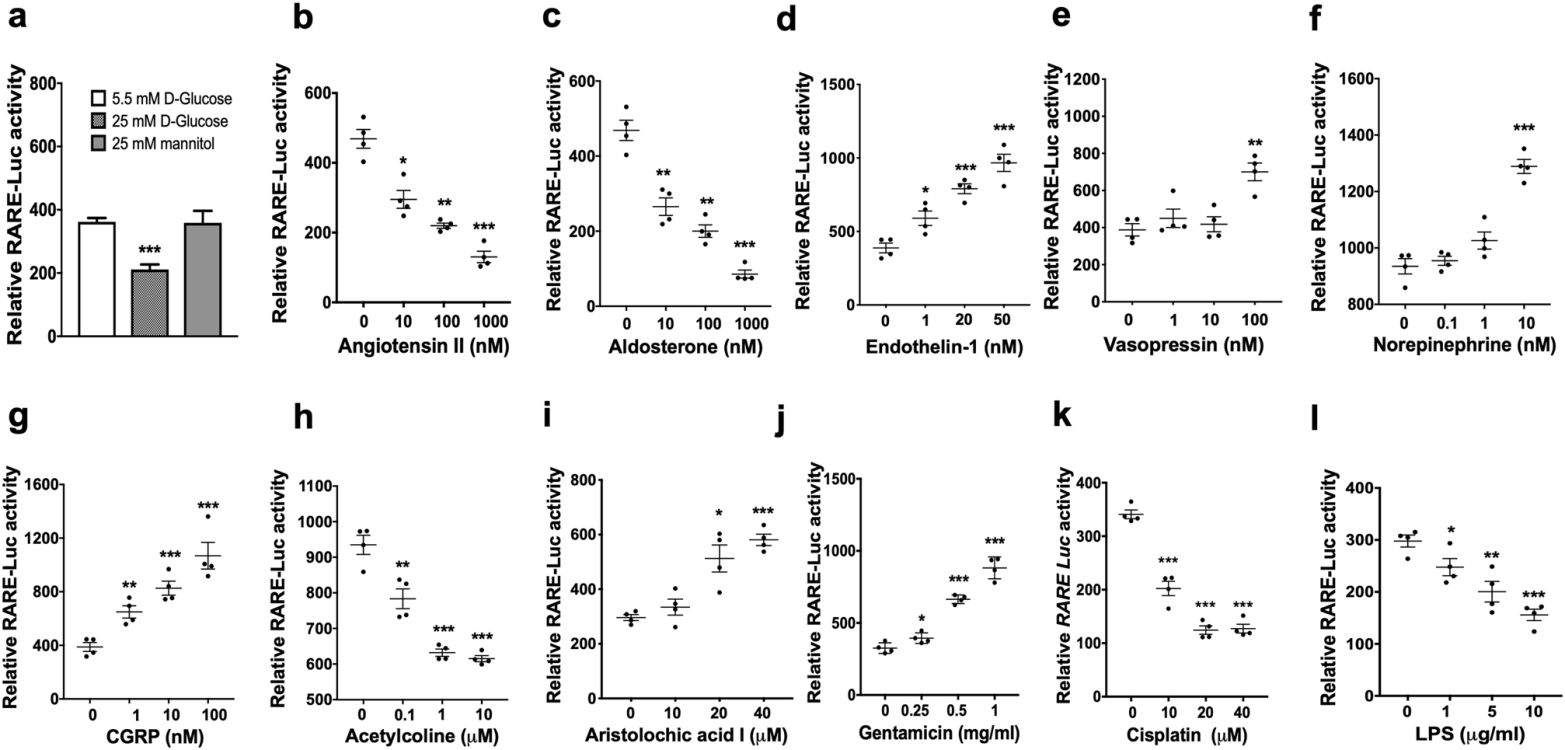
Neurotransmitters and mediators of kidney injury differentially regulated RA/RAR activity in mIMCD-3 cells. High-glucose treatment (but not 25 mM mannitol) for 24 h significantly reduced RA/RAR activity (**a**). In addition, 24 h exposure to angiotensin II (**b**), aldosterone (**c**), acetylcholine (**h**), cisplatin (**k**) and LPS (**l**) dose-dependently repressed, while endothelin-1 (**d**), vasopressin (**e**), norepinephrine (**f**), CRGP (**g**), aristolochic acid I (**i**) and gentamicin (**j**) dose-dependently induced RA/RAR activity. *, **, ***: *p* < 0.05, *p* < 0.01, *p* < 0.001 vs 5.5 mM D-Glucose or vehicle control groups.

Diabetes characterised by high blood and urinary glucose levels is a leading cause of CKD^52^. To examine whether high glucose may regulate RA/RAR activity, we compared the effects of 24 h exposure to 25 mM glucose (high glucose) and 5.5 mM glucose (normal medium control) on RA/RAR activity in mIMCD-3 cells, with 25 mM mannitol as an osmotic control for high-glucose treatment. We found that high glucose, but not equal molar concentrations of mannitol, significantly repressed RA/RAR activity (Fig. 4a).

### RA/RAR activity in CD cells was differentially regulated by mediators of hypertension and neurotransmitters

Hypertension is another major risk factor for CKD^53^. To examine whether mediators of hypertension may regulate RA/RAR activity in CD cells, we compared the effects of four well-established mediators of hypertension on RA/RAR activity in mIMCD-3 cells. We found that angiotensin II (0.01–1 μM) and aldosterone (0.01–1 μM) dose-dependently repressed, while endothelin-1 (1–50 nM) and vasopressin (100 nM) dose-dependently induced RA/RAR activity (Fig. 4b–e). More recently, renal nerves have been shown to contribute to hypertension^54,55^, AKI and its transition to CKD^56–59^. We then examined whether neurotransmitters norepinephrine, calcitonin gene-related peptide (CGRP) and acetylcholine may also regulate RA/RAR activity in CD cells. As shown in Fig. 4f,g, norepinephrine (10 nM) and CGRP (1–100 nM) activated, while acetylcholine (0.1–10 µM) repressed RA/RAR activity in CD cells (Fig. 4h). These results suggest that mediators of hypertension and neurotransmitters may have opposite effects on the RA/RAR activity in CD cells, apart from their systemic effects on blood pressure and organ perfusion. In view that angiotensin II and aldosterone are well-established mediators of CKD progression^3,4,53^, while vasopressin and norepinephrine are vasopressors often used to boost organ perfusion and prevent AKI in shock patients^60^, it is possible that differential regulation of RA/RAR activity in the CD may be a novel determinant in their differing roles in AKI and CKD, in addition to their systemic effects on blood pressure.

Of note, although we tested 0.1–10 nM norepinephrine and 1–100 nM vasopressin, only at the highest concentrations they induced RA/RAR activity. 10 nM norepinephrine is 4–20-fold higher than the plasma concentrations of healthy subjects^61^ and comparable with plasma concentrations of patients with phaeochromocytoma^61^ or those receiving norepinephrine as a vasopressor^62^. Vasopressin, however, primarily targets the renal CD. Thus, although its circulating concentrations in healthy subjects and patients are at picomolar levels^63,64^, it might be enriched at the CD and need higher local concentrations to regulate CD functions. In keeping with this notion, in mIMCD-3 cells, it took 10 nM vasopressin to activate the expression of the H^+^-K^+^-ATPase α_2_-subunit (HKα_2_) gene cells^65^ and 100 nM vasopressin was required to activate RA/RAR activity. Thus, both pathophysiological and pharmacological implications of our findings deserve further investigation.

### RA/RAR activity in CD cells was differentially regulated by nephrotoxic agents and other inducers of kidney injury

Depending on dosing regimens and duration of exposure, nephrotoxic agents aristolochic acid I and gentamicin, the chemotherapeutic drug cisplatin and endotoxin lipopolysaccharide (LPS) can induce AKI and/or CKD^1,66–69^. Although they are all broadly considered nephrotoxic, they are known to cause renal damage by different mechanisms^66^. For example, instead of direct cytotoxicity, cisplatin and LPS both induce AKI in a Toll-like receptor-4 (TLR4)-dependent manner^67,68^. As shown in Fig. 4i,j, aristolochic acid I (20–40 μM) and gentamicin (0.25–1 mg/ml) dose-dependently induced, while cisplatin (10–40 μM) and LPS (1–10 μg/ml) dose-dependently repressed RA/RAR activity in mIMCD-3 cells (Fig. 4k,l). Given the emerging evidence of a critical protective role for the RA/RAR activity in CD cells^9^, our results suggest that, LPS and cisplatin may disarm RA/RAR-mediated defensive mechanisms, leading to more progressive renal kidney injury, while activation of RA/RAR activity by aristolochic acid I and gentamicin may mount defence and mitigate injury.

### RA/RAR activity in autosomal recessive polycystic kidney disease (ARPKD) renal cystic cells was significantly lower than in age-matched normal CD cells

**Figure 5:**
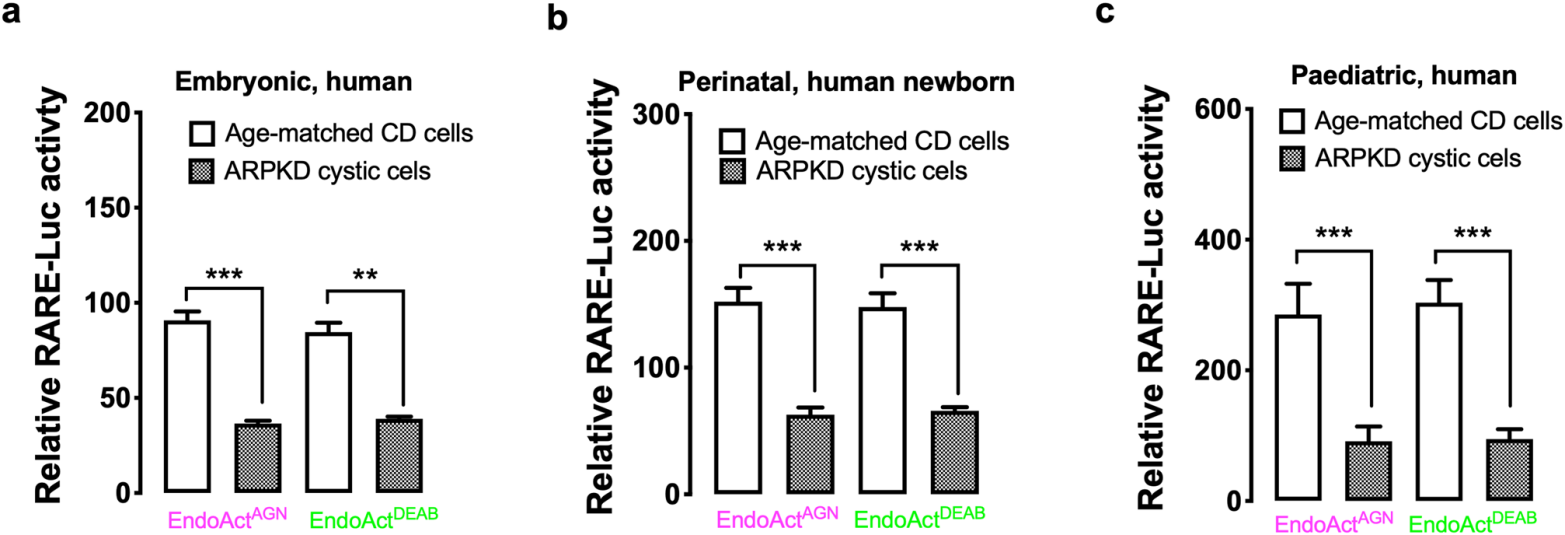
Endogenous RA/RAR activity in ARPKD renal cystic cells was significantly lower than age-matched normal CD cells. EndoAct^AGN^: Endogenous RA/RAR activity repressible by AGN193109; EndoAct^DEAB^: Endogenous RA/RAR activity repressible by DEAB. (**a**) embryonic; (**b**) perinatal; and (**c**) paediatric. **, ***: p < 0.01, 0.001, respectively.

In ARPKD, all renal cysts derive from the CD^70^. To examine whether RA/RAR activity in CD-derived ARPKD renal cystic cells is abnormal, we compared the endogenous RA/RAR activity in primary cultures of embryonic (foetal 24 weeks), perinatal (newborn) and paediatric (18 months old) ARPKD renal cystic cells and their age-matched normal human CD cells. Endogenous RA/RAR activity was calculated in two forms: (i) Endogenous RA/RAR activity repressed by 1 μM RAR antagonist AGN193109 (EndoAct^AGN^) = *RARE-Luc* activity (Vehicle) − *RARE-Luc* activity (AGN193109); (ii) Endogenous RA/RAR activity repressed by 25 μM of the RA biosynthesis inhibitor DEAB (EndoAct^DEAB^) = *RARE-Luc* activity (Vehicle) − *RARE-Luc* activity (DEAB). As shown in Fig. 5a–c, CD-derived ARPKD renal cystic cells consistently had significantly lower EndoAct^AGN^ and EndoAct^DEAB^ than age-matched normal CD cells. These data suggest that RA/RAR activity in ARPKD renal cystic cells may be reduced and the biological importance of this change in ARPKD deserves further investigation.

### RA-568 was more potent than atRA in rescuing RA/RAR activity repressed by albumin, aldosterone, angiotensin II, cisplatin, high glucose and LPS

**Figure 6:**
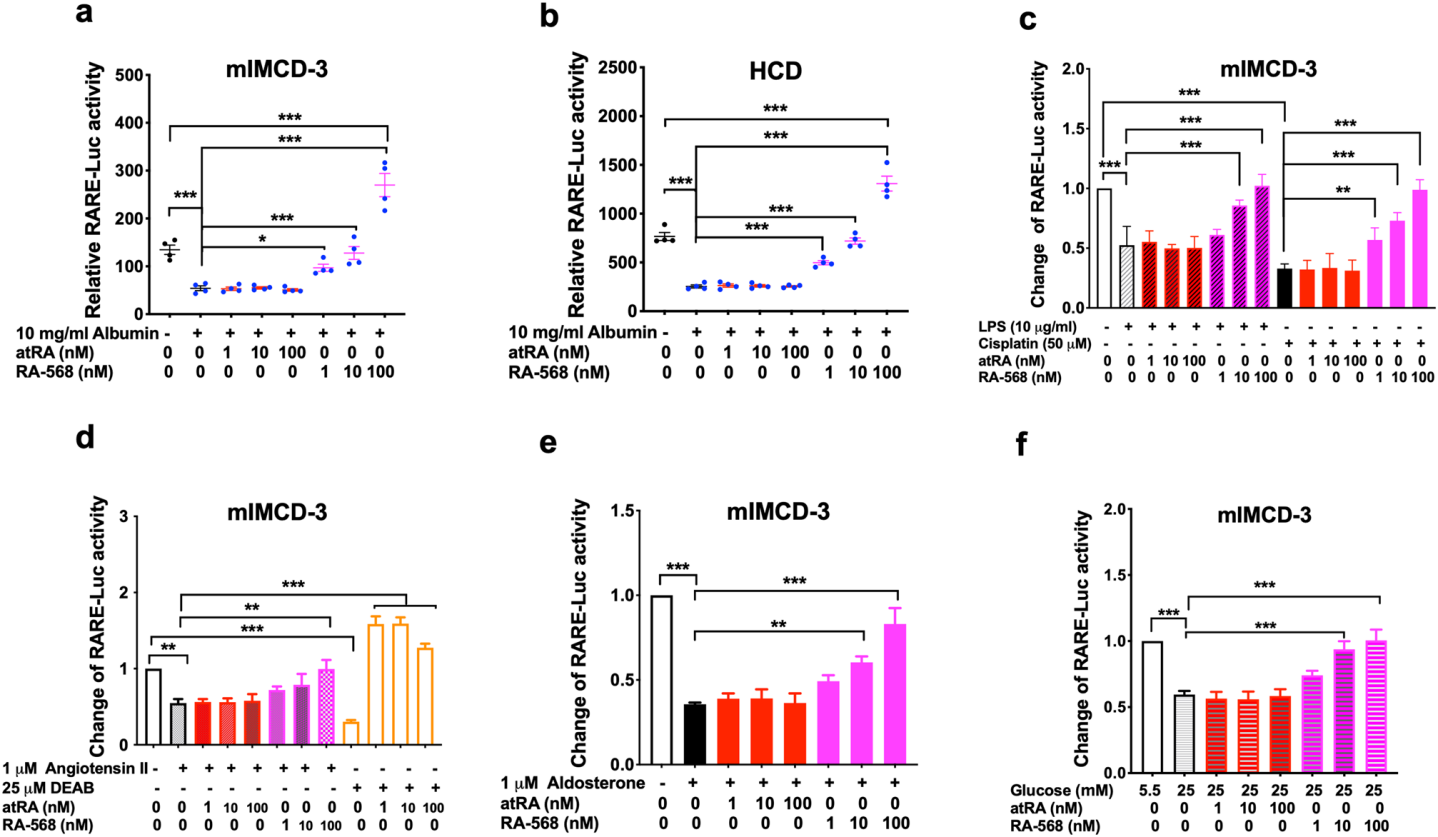
RA-568 was more potent than atRA in preventing RA/RAR repression by mediators of kidney injury. RA-568 (1–100 nM), but not atRA (1–100 nM) dose-dependently prevented RA/RAR repression by 24 h exposure to 10 mg/ml albumin (**a**,**b**), 50 μM cisplatin (**c**), 10 μg/ ml LPS (**c**), 1 μM angiotensin II (**d**), 1 μM aldosterone (**e**) and 25 mM glucose (**f**) in mIMCD-3 (**a**,**c**–**f**) and HCD cells (**b**). *, **, ***: *p* < 0.05, *p* < 0.01, *p* < 0.001, respectively.

Given the hypothesised protective role for the RA/RAR activity in the CD^9^, to prevent CKD and its progression, it may be desirable to prevent RA/RAR repression by albumin, aldosterone, angiotensin II, cisplatin, high glucose and LPS in these cells. To this end, we propose that the RARα agonist RA-568^41^ may be more suited than natural RAR pan-agonist atRA, because (i) it is structurally distinct from atRA (Supplementary Fig. 3), RA-568 may not be sequestered by albumin as much as atRA^40,45^; (ii) RA-568 activates RARα, but not RARβ and RARγ, nor RXR^41^, thus it will selectively activate RARα, the most abundant RAR isotype in CD cells^71^ and the kidney (Supplementary Fig. 1), while minimising RARγ-mediated irritating skin syndrome^19^ and RXR-mediated pro-fibrotic effects of atRA metabolites^72^. Indeed, in both mouse (mIMCD-3) and human (HCD) CD cell lines, we found that RA-568 was at least 100-fold more efficacious than atRA in restoring RA/RAR activity repressed by 10 mg/ml albumin. Though not totally unexpected, it was striking to find that atRA at concentrations as high as 100 ng/ml had no effect on the RA/RAR activity repressed by 10 mg/ml albumin. This was likely because high-concentration albumin sequesters both endogenous and exogenous atRA. In contrast, in both mIMCD-3 mouse CD cells and HCD human CD cells, RARα agonist RA-568 efficaciously restored RA/RAR activity repressed by 10 mg/ml albumin. At 1–10 nM, it restored RA/RAR loss of activity; and at 100 nM the RA/RAR activity was significantly higher than the vehicle control group (Fig. 6a,b). Further, as shown in Supplementary Fig. 5, RA/RAR activity induced by 100 nM RA-568 in both mIMCD-3 and HCD cells was not repressed by increasing concentrations of albumin (0.3–10 mg/ml), in contrast to 100 nM atRA, which neither boosted RA/RAR activity nor prevented RA/RAR activity from being repressed by albumin in a dose-dependent manner (1–10 mg/ml). These data suggest that selectively activating RARα by RA-568 is likely a better approach than pharmaceutical atRA to restoring and boosting RA/RAR activity in CD cells.

We next examined the effects of atRA and RA-568 in preventing RA/RAR repression by LPS and cisplatin in mIMCD-3 cells. As shown in Fig. 6c, RA/RAR activity repression by either LPS (10 μg/ml) or cisplatin (50 μM) was not affected by 1–100 nM atRA but was significantly and dose-dependently prevented by RA-568, at 10–100 nM and 1–100 nM, respectively. Similarly, we compared effects of atRA and RA-568 in preventing RA/RAR repression by angiotensin II, aldosterone and high glucose. As shown in Fig. 6d–f, RA/RAR repression by either angiotensin II, aldosterone or high glucose in mIMCD-3 cells was prevented by 100 nM RA-568; 10 nM RA-568 also effectively prevented RA/RAR activity repression by aldosterone and high glucose. In contrast, 1–100 nM atRA did not prevent angiotensin II, aldosterone and high glucose from repressing RA/RAR activity. The ineffectiveness of atRA in all these settings was in stark contrast to the excellent efficacy of 1–100 nM atRA in preventing RA/RAR repression by 25 μM DEAB, an inhibitor of RA synthesis (Figs. 2a, 6d)^21^.

## Discussion

AKI and CKD are multifactorial disorders^2–4,7^. This study highlights the RA/RAR activity in CD cells as a convergence point of regulation by the multiple factors involved in AKI and CKD. Intriguingly, it responds in an opposite fashion to mediators of kidney injury (induced by gentamicin and aristolochic acid I; repressed by cisplatin and LPS), mediators of hypertension (induced by endothelin-1, vasopressin and norepinephrine; repressed by aldosterone and angiotensin II) and neurotransmitters (induced by CGRP and norepinephrine; repressed by acetylcholine). Further investigations into the mechanisms and biological consequences of RA/RAR activity changes induced by these factors may facilitate deeper understanding of the heterogeneity and multifactorial nature of AKI and CKD and guide refined therapies.

In particular, this report establishes that RA/RAR activity responds differentially to a wide range of neurotransmitters and mediators of kidney injury and thus is unlikely a bystander in AKI and CKD. This rationalise the next step of studies to test the exact roles for the RA/RAR in the CD in different AKI and CKD models, e.g. by selectively up- and down-regulating RA/RAR activity and its downstream genes in the CD cell lineage using *Hoxb7* or *AQP*2 promoters and then examining whether this changes the outcomes of AKI and CKD models in mice^9^.

The main limitation of this work is that evidence we have presented so far is mainly from in vitro models. Although our in vitro and in vivo data both support the capacity of heavy albuminuria to repress RA/RAR activity in CD cells (Fig. 1, 2, 3), we are acutely aware that regulation of the RA/RAR pathway in the CD must be more complex in vivo than in cultured CD cells, and eventually, in vitro, in vivo and clinical studies must be analysed together to fully understand the regulation of the RA/RAR pathway in the CD. For example, high glucose has been reported to repress RAR expression in mIMCD-3 cells^71^. Thus, reduced RAR expression may, at least in part, contribute to high-glucose repression of RA/RAR activity in mIMCD-3 cells. In diabetic mouse kidneys, atRA biosynthesis and atRA-dependent gene expression are compromised^73^, at least in part, due to o-GlcNAcylation of proteins crucial for retinoid binding, metabolism and signalling^74^. Furthermore, a biphasic regulation of renal RA/RAR signalling has been suggested by transcriptomic analysis of renal biopsy tissues of early- and late-stage diabetic nephropathy, respectively^75^.

Similar to our findings in mIMCD-3 cells, LPS has been previously reported to repress RA/RAR signalling in hepatic stellate cells^76^ and gentamicin has been reported to induce RA/RAR activity in renal tubules in zebra fish^39^. Given the potential importance of RA/RAR signalling in the CD^9^ and the potential differences in vitro and in vivo, it is desirable to clarify how renal RA/RAR activity is regulated in different types of AKI and CKD in RARE-reporter animals^21,22,39,40^ and in related clinical settings. To know exactly how this pathway is regulated will pave the way for further mechanistic studies, which will guide the development of intervening strategies, if indicated.

Taking ARPKD, a disease of the focal adhesion complex and the primary cilia due to mutations of fibrocystin (encoded by *PKHD1*)^70^, for example, we found that ARPKD renal cystic cells had significantly lower endogenous RA/RAR activity than age-matched control CD cells. Although we have three age-matched pairs leading to exactly the same findings, larger sample size may be needed to further confirm our findings. To examine a possible causal relation, it may be necessary to examine whether genetic mutations of *PKHD1* in normal CD cells repress RA/RAR signalling and, if so, its role in ARPKD pathogenesis. Based on RA/RAR-dependent gene profiling in CD cells^21^, we hypothesise that decreased RA/RAR activity in CD cells may reduce expression of focal adhesion complex components tensin (*Tns1*) and α2-integrin (*Itga2*), and the cilium-specific transcription factor Foxj1 (*Foxj1*), impair signalling through focal adhesion complexes and cilia, leading to disease progression. If this is true, restoring the RA/RAR activity in the CD cell lineage may curb progression of this devastating disease and becomes a cost-effective strategy to improve ARPKD prognosis.

The inefficacy of atRA in preventing RA/RAR repression by albumin, high glucose, cisplatin, LPS, aldosterone and angiotensin II was an important finding. Since in parallel studies atRA was always highly efficacious in preventing RA/RAR repression by the atRA biosynthesis inhibitor DEAB, RA/RAR repression by the aforementioned mediators of kidney injury may not be simply due to lack of the bioactive ligand atRA. Thus, further mechanistic studies are clearly needed to guide development of both retinoid- and non-retinoid-based rescuing strategies. The good news is that, in contrast to the poor efficacy of atRA, the synthetic RARα agonist RA-568 has shown excellent effect in preventing RA/RAR repression. As this compound has an established safety profile in preclinical studies in mice and rats^41^, it could be an ideal drug lead for AKI and/or CKD, by boosting or restoring RA/RAR activity in kidneys, particularly in the CD. Thus, our in vitro data justify further translational studies comparing the efficacy and safety of RA-568 and atRA as potential renal therapeutics.

Despite the focus on CD cells of the present study, we acknowledge the possibility that RA/RAR activity in other renal cell types could be activated in pathological conditions. If so, how renal RA/RAR activity pathologically re-distributes in different AKI and CKD, whether such pathologically activated signalling in other renal cells, such as proximal tubular cells and infiltrating cells in ischaemia–reperfusion AKI^39^ and renal progenitor cell in Adriamycin-induced nephropathy^40^, has the same roles as the RA/RAR in the CD, and how all these RA/RAR signalling interacts are important questions to answer. Eventually, all such knowledge should need be integrated to fully understand the pathological roles for the RA/RAR signalling in AKI and CKD and to devise intervention strategies**.**

In conclusion, we have found that RA/RAR activity in cultured CD cells responds to a wide range of neurotransmitters and mediators of kidney injury, either being induced or repressed. When repressed, the activity is poorly rescued by exogenous atRA, but is effectively reversed by RARα agonist RA-568. Further studies revolving around this newly found convergent point of regulation in AKI and CKD may help us better understand these interconnected syndromes and lead to novel therapeutics.

## Methods

### Studies on animal tissues

#### Immunofluorescence confocal microscopy

No new animal study was performed in this study. Instead, we performed confocal microscopy (LSM510 META, Carl Zeiss, Jena, Germany) on 10 μm frozen sections of renal tissues of *RARE-LacZ* mice (CD1 background, 6- to 10-week-old, female) collected in our previously published project, including nine with trace albuminuria at dip-stick tests (approximately 0.1–0.2 mg/ml albumin or albumin:creatinine ratio < 0.3) and six with high levels of albuminuria, with albumin:creatinine ratios of > 7 (17.7 ± 2.99), induced by two retro-orbital injections of 14 mg/kg Adriamycin in phosphate-buffered saline (PBS), as well as five healthy controls treated by two successive injections of PBS^40^. Animal experiments were approved by the University of Florence Animal Studies Review Board, performed in accordance with institutional, regional, and state guidelines, and adhered to the National Institutes of Health Guide for the Care and Use of Laboratory Animals. Dolichos biflorus agglutinin (DBA)-lectin (Vector Laboratories, Peterborough, UK) was used to stain CD cells. Chicken anti–β-galactosidase polyclonal antibody (Abcam, Cambridge, UK) and AlexaFluor 488-labelled donkey anti-chicken antibody (Jackson ImmunoResearch, West Grove, PA) were used to stain *LacZ*-expressing cells and Topro-3 (Molecular Probes, Invitrogen, Carlsbad, CA) was used for nuclear staining. Sections were also stained with secondary antibodies only to serve as negative control, as we previously reported^40,77^. To quantify the number of *LacZ*-expressing cells in the CD, β-gal- and DBA-lectin-positive cells were counted in at least 4 renal cortex sections of each mouse and percentage of dual positive cells was calculated. Five fields were counted on each section.

### Cell biology studies

#### Reagents

Acetylcholine, AGN193109, aldosterone, aristolochic acid I, cisplatin, CGRP, DEAB, dexamethasone, D-glucose, endothelin-1, gentamicin, human albumin, human angiotensin II, human IgG, human transferrin, LPS, mannitol, norepinephrine, tetracycline, atRA and vasopressin were purchased from Sigma-Aldrich Company Ltd, Gillingham, UK. RA-568 was synthesised by Sygnature Discovery, Nottingham, UK. All reagents dissolved in ethanol and/or dimethylsulphoxide (DMSO) were first diluted to 1000 × stock solution and then diluted 1000 × with culture medium to the working concentrations. Control groups were treated with 0.1% ethanol and/or DMSO.

#### Cell cultures

mIMCD-3 and M-1 cells (LGC Standards, Middlesex, UK) and mPTEC cells (kind gift by Dr Mark Dockrell, South West Thames Institute for Renal Research, Surrey, UK) originally derived from the inner medullary CD, cortical CD and the S3 segment of the proximal tubule of a Brinster transgenic mouse [Tg(SV40E)Bri7], respectively^24,48,78^. They were routinely grown in DMEM-F12 medium containing 100 μg/ml penicillin and 100 μg/ml streptomycin (thereafter known as antibiotics) (PAA Laboratories Ltd, Somerset, UK), supplemented with 5% foetal calf serum (FCS; Life Technologies Ltd, Paisley, UK). Both mIMCD-3 and M-1 cells were 100% positive for aquaporin-2 (AQP2), a CD principal cell marker, and mPTEC cells were 100% positive for proximal tubular cell marker aquaporin-1 (AQP1). NRK-52E and NRK-49F cells (LGC Standards) are epithelial and fibroblast-like cell clones from kidneys of an adult non-inbred Osborne-Mendel rat^79^. They were routinely grown in 5% FCS DMEM with antibiotics (PAA). NRK-49F cells stained positive for vimentin and negative for cytokeratin and factor VIII. RNAs extracted from M-1, mIMCD-3 and mPTEC were selectively amplified using mouse specific primers, while RNA extracted from NRK-49F cells was selectively amplified using rat specific primers only. HCD cells are a well-characterised human cortical CD cell line^50^ provided by Dr Remi Piedagnel (INSERM, Paris, France). They were cultured in 2% FCS DMEM-F12 with antibiotics and insulin-transferrin-selenium supplement (ITS) (PAA). Mouse CD-derived MSCs (Hoxb7 B2) were provided by Dr Joan Li (University of Queensland, Queensland, Australia) and fully characterised as reported before^49^. They were cultured in 20% FCS α-MEM medium with antibiotics (PAA). Primary human CD cells and their age-matched embryonic (24 weeks), newborn and paediatric (18-month old) ARPKD renal cystic cells were fully characterised by Professor Patricia Wilson^80–82^ and were obtained from the Polycystic Kidney Disease Charity Biobank. Both foetal and paediatric ARPKD renal CD cells and their age-matched normal CD cells were routinely grown in 3% FCS 50% Click’s medium: 50% RPMI 1640 media (Gibco, Life Technologies) supplemented with antibiotics and 5 µg/ml human transferrin, 5 × 10^–8^ M dexamethasone, 2 mM GlutaMax (Gibco). Cells were plated in 6-well plates coated with 4.1 mg/ml collagen type-I (Corning, NY) and left to attach overnight before further studies. All cell cultures were maintained at 37 °C and 5% CO_2_.

#### Transient transfection and RARE dual luciferase assays

Three tetracycline-inducible gene expression plasmids were procured from VectorBuilder, Santa Clara, CA for transient transfection studies: (i) *ALB-EGFP,* a plasmid with DNA inserts of enhanced green fluorescence protein (EGFP) and the wild-type *ALB* gene (Accession NM_000477.5); (ii) mutant *ALB-EGFP*, a plasmid with DNA inserts of *EGFP* and mutated *ALB* genes, encoding human albumin deleted of amino acids 139–189, including 11 of 12 amino acids critical for binding atRA^45^; and (iii) a control vector, with the EGFP DNA insert only. The maps of these plasmids are shown in Supplementary Fig. 6. Lipofectamine LTX, Plus reagents and Opti-MEM I Reduced Serum Medium (Gibco) were used in transient transfection of plasmid DNAs according to the manufacturer’s instructions.

For RARE dual luciferase assay, pGL3-RARE-luciferase (https://www.addgene.org/13458/)^83^ and PRLSV40 (https://www.addgene.org/vector-database/3949/) plasmids (Promega, Southampton, UK) were used to co-transfect cells at a ratio of 5:1 to report RARE-dependent expression of Firefly luciferase and constitutive expression of Renilla luciferase. Cells were lysed with Reporter Lysis Buffer (Promega) and luminescence signal was detected with Luciferase Assay System and Beta-Glo Assay System (Promega). “Background signal” was determined as the average readout from wells treated with transfection medium only without any plasmid DNA and this was subtracted from readouts of other wells. The reporter assay results were expressed as relative unit (RLU, ratios of readouts of the two reporters). Alternatively, RLU of treatment group was normalised to vehicle control group and expressed as fold-change. Because pGL3-RARE-Luc and the control PRLSV40 plasmid both have the SV40 minimal promoter, the RLU (ratios of readouts of the two reporters) always comprises two parts, one dependent on RA/RAR and RARE (RA/RAR activity) and the rest being “baseline signal” mediated by the minimal promoter in the pGL3-RARE-Luc plasmid. The expected effects of DEAB and AGN193109 on the reporter assay are as follows: (i) DEAB represses RA/RAR activity induced by endogenously synthesised RA but does not repress the effects of the trace amount of RA in 1% FCS in the culture medium and any exogenous RAR agonists, if applicable, nor does it repress any RA-independent transcription activity of RAR mediated by the AF-2 domain of the nuclear receptor^84^; (ii) AGN193109 is not only a potent pan-RAR antagonist (antagonising effects of RAR agonists and inhibiting RA/RAR-mediated AF1-mediated gene expression) but also a RAR inverse agonist (actively recruiting corepressors to RAR and thus repressing RAR agonist-independent AF2-mediated transcriptional activity of RAR)^84^. At the concentrations used in this study, both DEAB and AGN193109 almost completely switched off RA/RAR-dependent *Dhrs3*, *Sprr1a* and *Ppbp* mRNA expression, but AGN193109 was consistently more potent in pressing these gene expression (− 99%, − 95% and − 93%) than DEAB (− 96%, − 85% and − 86%)^23^. Thus, DEAB incomplete repression of RAR activation by endogenous RA, DEAB inability to repress effects of RA in 1% FCS cell culture medium and AGN193109 incomplete repression of RA/RAR activity may not play any major role in the RLU values unrepressed by DEAB and AGN193109 in the RARE dual luciferase results in this project, although some minor roles cannot be completely ruled out. Nonetheless, this study particularly focuses on endogenous RA/RAR activity, which is defined as net RLU values repressed by both DEAB and AGN193109.

#### Experimental protocols

For dual luciferase reporter assays, mIMCD-3, HCD, M-1, Hoxb7 B2 MSCs, mPTEC, NRK-49F and NRK-52E cells were seeded into 24-well plates at 4 × 10^4^ cells/well in 500 μl of the corresponding complete medium with FCS but without antibiotics. After culturing for 16 h, cells were co-transfected with pGL3-RARE-luciferase and PRLSV40 plasmids and cultured for another 6 h. Medium was then changed to 1% FCS fresh medium. After an overnight incubation, medium was changed to 1% FCS fresh medium containing 1 μM AGN193109, 25 μM DEAB, 10 mg/ml albumin and cultured for 24 h. mIMCD-3 cells were also treated for 24 h with albumin ranging from 0.3 to 10 mg/ml, 10 mg/ml IgG, 10 mg/ml transferrin, angiotensin II (0.01–1 μM), aldosterone (0.01–1 μM), 5.5 mM and 25 mM D-glucose, 25 mM mannitol, LPS (1–10 μg/ml), cisplatin (10–40 μM), vasopressin (1–100 nM), CGRP (1–100 nM), endothelin-1 (1–50 nM), gentamicin (0.25–1 mg/ml), aristolochic acid I (10–40 μM), norepinephrine (0.1–10 nM) and acetylcholine (0.1–10 μM).

In some studies, RARE dual luciferase reporter assays were conducted in mIMCD-3 and HCD cells cultured in 96-well plates. The cells (5 × 10^3^/well) were seeded in 200 μl of culture medium without antibiotics. Following medium changes and transient transfection same as above, effects of albumin (0.3–10 mg/ml), with and without atRA (100 nM) or RA-568 (100 nM), and of albumin (10 mg/ml), DEAB (25 μM), high glucose (25 mM), angiotensin II (1 μM), aldosterone (1 μM), LPS (10 μg/ml) or cisplatin (50 μM), with or without 1–100 nM atRA or RA-568 for 24 h, on RA/RAR activity was analysed.

For *ALB* overexpression studies, mIMCD-3 cells were seeded into a 24-well plate at 4 × 10^4^ cells/well in 500 μl 5% FCS-DMEM-F12 without antibiotics. After culturing for 16 h, cells were co-transfected with tetracycline-inducible *ALB-EGFP,* mutant *ALB-EGFP* or the *EGFP*-only control plasmid, together with pGL3-RARE-luciferase and PRLSV40 plasmids for 6 h. Medium was then changed to 1% FCS DMEM-F12. After an overnight incubation, medium was changed to 1% FCS DMEM-F12 supplemented with 2 μg/ml tetracycline for 24 h to switch on transgenes.

For reporter assays involving primary human CD cells and age-matched ARPKD renal cystic cells, cells were seeded into 96-well plates, 3.5 × 10^4^/well, in 500 μl 3% FCS 50% Click’s medium: 50% RPMI 1640 without antibiotics. After culturing to about 70% confluence, cells were co-transfected with pGL3-RARE-luciferase and PRLSV40 plasmids and cultured for another 6 h. Medium was then changed to fresh 1% FCS medium. After an overnight incubation, medium was changed to 1% FCS medium with 1 μM AGN193109, 25 μM DEAB, 10 mg/ml albumin or vehicle and cultured for 24 h.

### Data analysis

Immunofluorescence confocal microscopy data are expressed as percentage of *LacZ*-expressing DBA-positive cells over total DBA-positive cells per renal cortex section. All cell biology experiments were performed in quadruplicate wells for each group, with reproducible results in at least two independent experiments. Results are presented as mean ± SEM. For comparison of two and more than two groups, t-tests (non-paired, two-tail; with Welsh’s correction if any data of comparison failed Shapiro–Wilk normality tests) and one-way ANOVA analysis (including Tukey’s multiple comparisons) were conducted, respectively. For comparison of fold-changes, data were log-transformed before statistical analysis. Statistical significance was determined as *p* < 0.05. Analyses were performed using GraphPad Prism, Version 8.0 (GraphPad Software Inc., CA).

## Supplementary information

Supplementary file1
