# Supplementary material for: Collecting duct cells show differential retinoic acid responses to acute versus chronic kidney injury stimuli
Source: Sci Rep. 2020 Oct 7;10:16683. doi: 10.1038/s41598-020-73099-9 (PMC7542174; doi:10.1038/s41598-020-73099-9)
Supplement: Supplementary file 1 — Supplementary file1 [file 41598_2020_73099_MOESM1_ESM.docx]

**Collecting duct cells show differential retinoic acid responses to acute versus chronic kidney injury stimuli**

Alexandros Papadimitriou**^1^**, Paola Romagnani**^2^**, Maria Lucia Angelotti^2^, Mazhar Noor**^1^**, Jonathan Corcoran**^3^**, Katie Raby**^4^**, Patricia D Wilson**^4^**, Joan Li**^5^**, Donald Fraser**^6^**, Remi Piedagnel**^7^**, Bruce M Hendry**^1^** and Qihe Xu**^1,*^**

**1**. Renal Sciences and Integrative Chinese Medicine Laboratory, Department of Inflammation Biology, School of Immunology & Microbial Sciences, Faculty of Life Sciences & Medicine, King’s College London, London, UK;

**2.** Department of Clinical and Experimental Biomedical Sciences, University of Florence, Florence, Italy;

**3.** The Wolfson Centre for Age-Related Diseases, King's College London, London, UK;

**4.** University College London, UCL Centre for Nephrology, Royal Free Hospital, London, UK;

**5.** Faculty of Medicine, University of Queensland, Brisbane, Queensland, Australia;

**6.** Wales Kidney Research Unit, Heath Park Campus, Cardiff, UK;

**7.** National Institute for Health and Medical Research (INSERM), Unité Mixte de Recherche (UMR)-S1155, Tenon Hospital, Sorbonne Universités, Paris, France

*. Corresponding author: [qihe.xu@kcl.ac.uk](mailto:qihe.xu@kcl.ac.uk)

**Supplementary Figure 1. RAR mRNA expression in kidney and liver of C57Bl/6 mice.** Adapted from doi: 10.1621/datasets.02001.

**Supplementary Figure 2. Molecular structures of atRA and RA-568**

**
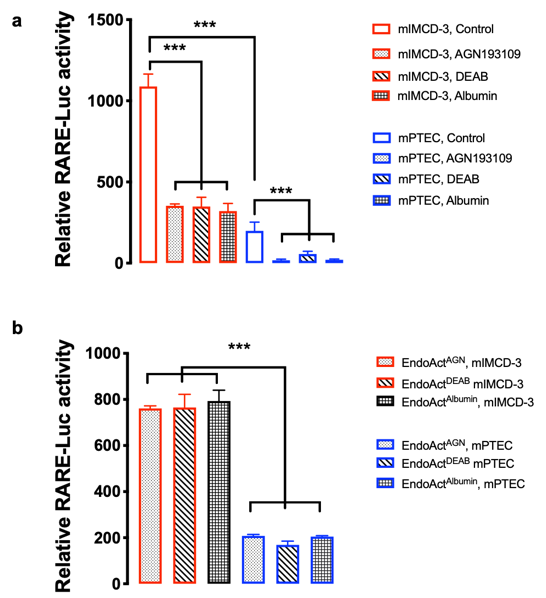
**

**Supplementary Figure 3. Comparison of *RARE-Luc* reporter activities in mIMCD-3 and mPTEC cell lines.** mIMCD-3 and mPTEC cells were subjected to RARE dual luciferase assay, in the presence of 1 μM AGN193109, 25 μM DEAB, 10 mg/ml albumin or vehicle only for 24 h. In both mIMCD-3 and mPTEC cells, AGN193109, DEAB and albumin significantly repressed gross basal RARE-luciferase activity **(a)**. Endogenous RA/RAR activities repressible by AGN193109 (EndoAct^AGN^), DEAB (EndoAct^DEAB^) and albumin (EndoAct^Albumin^) in mIMCD-3 and mPTEC cells were calculated and displayed in three forms (**b**): (i) Endogenous RA/RAR activity repressed by 1 μM RAR antagonist AGN193109 (EndoAct^AGN^) = *RARE-Luc* activity (Vehicle) - *RARE-Luc* activity (AGN193109); (ii) Endogenous RA/RAR activity repressed by 25 μM the RA biosynthesis inhibitor DEAB (EndoAct^DEAB^) = RARE-Luc activity (Vehicle) - RARE-Luc activity (DEAB); (iii) Endogenous RA/RAR activity repressed by 10 mg/ml albumin (EndoAct^Albumin^) = *RARE-Luc* activity (Vehicle) - *RARE-Luc* activity (Albumin). ***: *p*<0.001.


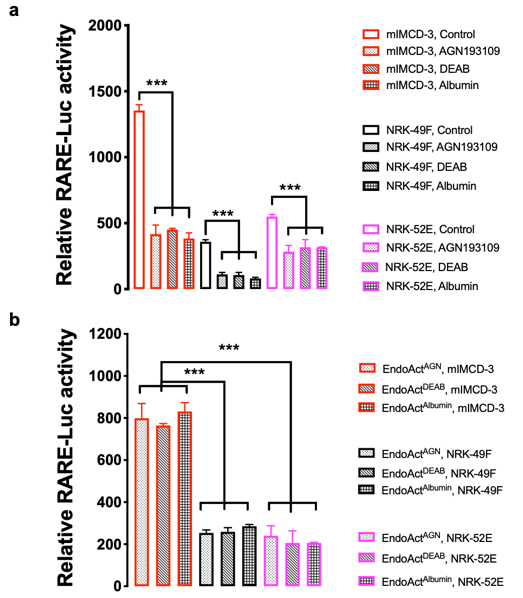


**Supplementary Figure 4. Comparison of *RARE-Luc* reporter activities in mIMCD-3, NRK-52E and NRK-49F cell lines.** mIMCD-3, NRK-52E and NRK-49F cells were subjected to RARE dual luciferase assay, in the presence of 1 μM AGN193109, 25 μM DEAB, 10 mg/ml albumin or vehicle only for 24 h. In all these cell lines, AGN193109, DEAB and albumin significantly repressed gross RARE-luciferase activity (**a**). Endogenous RA/RAR activity repressible by AGN193109 (EndoAct^AGN^), DEAB (EndoAct^DEAB^) and albumin (EndoAct^Albumin^) in mIMCD-3, NRK-49F and NRK-52E cells are shown in **b**. ***; p<0.001.

**Supplementary Figure 5. Effects of 100 nM atRA and RA-568 on RA/RAR activity in mouse and human CD cells, with or without increasing concentrations of albumin.** mIMCD-3 cells treated by vehicle, 100 nM atRA or 100 nM RA-568, with and without 0.3, 1, 3 and 10 mg/ml albumin were subjected to RARE dual luciferase assay. RA-568, but not atRA, significantly increased basal RA/RAR activity; 1-10 mg/ml albumin dose-dependently repressed RA/RAR activity despite 100 nM atRA treatment, but it did not repress RA/RAR activity in the presence of 100 nM RA-568 (**a**). Similar results were observed in HCD cells (**b**). *, **, ***: *p*<0.05, *p*<0.01 *p*<0.001, respectively.

**Supplementary Figure 6. Maps of control vector (a) and the plasmids for tetracycline-inducible expression of wild-type (b) and mutant human *ALB* genes (c).**
